# Inducing Chronic Excitotoxicity in the Mouse Spinal Cord to Investigate Lower Motor Neuron Degeneration

**DOI:** 10.3389/fnins.2016.00076

**Published:** 2016-03-02

**Authors:** Catherine A. Blizzard, K. M. Lee, Tracey C. Dickson

**Affiliations:** Menzies Institute for Medical Research, University of TasmaniaHobart, TAS, Australia

**Keywords:** lower motor neuron, neurodegeneration, excitotoxicity, *in vivo* models, motor neuron disease

## Abstract

We report the methodology for the chronic delivery of an excitotoxin to the mouse spinal cord via surgically implanted osmotic mini-pumps. Previous studies have investigated the effect of chronic application of excitotoxins in the rat, however there has been little translation of this model to the mouse. Using mice that express yellow fluorescent protein (YFP), motor neuron and neuromuscular junction alterations can be investigate following targeted, long-term (28 days) exposure to the α-Amino-3-hydroxy-5-methyl-4-isoxazolepropionic acid (AMPA) receptor excitotoxin, kainic acid. By targeting the L3-4 region of the lumbar spinal cord, with insertion of an intrathecal catheter into the subarachnoid space at L5, chronic application of the kainic acid results in slow excitotoxic death in the anterior ventral horn, with a significant (*P* < 0.05) reduction in the number of SMI-32 immunopositive neurons present after 28 days infusion. Use of the Thy1-YFP mice provides unrivaled visualization of the neuromuscular junction and enables the resultant distal degeneration in skeletal muscle to be observed. Both neuromuscular junction retraction at the gastrocnemius muscle and axonal fragmentation in the sciatic nerve were observed after chronic infusion of kainic acid for 28 days. Lower motor neuron, and distal neuromuscular junction, degeneration are pathological hallmarks of the devastating neurodegenerative disease Amyotrophic Lateral Sclerosis (ALS). This mouse model will be advantageous for increasing our understanding of how the pathophysiological phenomena associated with this disease can lead to lower motor neuron loss and distal pathology, as well as providing a robust *in vivo* platform to test therapeutic interventions directed at excitotoxic mechanisms.

## Introduction

Excitotoxicity is a common pathogenic mechanism in many neurodegenerative diseases and may play an important initiating role in amyotrophic lateral sclerosis (ALS) (Vucic and Kiernan, 2009; Mehta et al., 2013). ALS is the most common form of motor neuron disease and one of the most devastating neurodegenerative disorders (Talbot, 2014), with patients dying within 2–5 years of diagnosis from respiratory failure. Gene mutations affecting several proteins, including TDP-43, FUS, c9orf72, and SOD1 have been identified in familial forms of the disease, however, the majority of cases appear sporadic (Kiernan et al., 2011; Ajroud-Driss and Siddique, 2015). ALS results in muscle paralysis caused by the degeneration of the motor neurons and neuromuscular junctions in the cortico-motor-neuronal system, however it is not clear why these neurons are selectively vulnerable. Although it is likely that ALS is a multifactorial disease, potentially involving RNA dysfunction, protein misfolding and aggregation, autophagy, ER stress and axonal disruption (Gibson and Bromberg, 2012), there is increasing evidence for a pivotal role for excitotoxicity in the pathogenesis, with a significant period of neuronal dysfunction occurring prior to frank cell loss (Corona et al., 2007; Kiernan et al., 2011). Indeed, hyperexcitability of both upper and lower motor neurons has been reported prior to the onset of motor symptoms(Pieri et al., 2003; Vucic and Kiernan, 2006; van Zundert et al., 2008; Menon et al., 2015) and the only current treatment available for ALS, riluzole, involves inhibition of this excitotoxic pathway (Miller et al., 2012).

Previous research has demonstrated that motor neurons are selectively vulnerable to α-amino-3-hydroxy-5-methyl-4-isoxazolepropionic acid (AMPA) mediated excitotoxicity (Vandenberghe et al., 2000; King et al., 2007). To explore this AMPA mediated mechanism of neurodegeneration, a rat model of site-specific application of excitotoxins to the spinal cord was developed (Nakamura et al., 1994; Kwak and Nakamura, 1995; Hirata et al., 1997; Sun et al., 2006). This model demonstrated that exposing the spinal cord to an excitotoxin, specifically an AMPA agonist, leads to significant motor neuron loss and replicates the loss of motor function that is characteristic of the human disease (Nakamura et al., 1994; Kwak and Nakamura, 1995; Hirata et al., 1997; Sun et al., 2006). These previous studies were restricted to analysis of neuropathology in the spinal cord and resulting motor dysfunction. Despite the continued implication of excitotoxicity in the pathobiology of ALS, the use of this model has been limited.

Here, we report the methodology required to achieve chronic exposure of the mouse lumbar region of the spinal cord to kainic acid, over a 28 day time course, and the resulting localized excitotoxic death of motor neurons. By modifying both the region of the spinal cord targeted, and the entry point of the delivery catheter, chronic application of toxins can be achieved without causing physical damage to the spinal cord. The application of this protocol to the Thy1-YFP mouse, first described by Feng and colleagues (Feng et al., 2000), extends the utility of this model. Yellow fluorescent protein (YFP) mice express YFP ubiquitously in all lower motor neuron axons and presynaptic terminals. Therefore, by establishing a chronic model of slow excitotoxic motor neuron death in the YFP mouse the distal axonal and neuromuscular junction pathology can now be investigated in conjunction with assessments of proximal cell body pathology and motor neuron subtype vulnerability. With the ability to investigate complete neuromuscular junction integrity and track their changes over time we have previously used this model to demonstrate that lower motor neurons have an underappreciated capacity for plasticity prior to neuronal degeneration (Blizzard et al., 2015). This model can be utilized to both increase our understanding of the mechanisms that lead to the motor neuron loss and neuromuscular junction degeneration that characterizes neurodegenerative conditions such as ALS and also as a platform for testing new therapeutic interventions targeted at lower motor neurons.

## Materials and methods

All experiments were approved by the Animal Ethics Committee of the University of Tasmania and are in accordance with the NHMRC Australian Code of Practice for the Care and Use of Animals for Scientific Purposes, 2013. Thy-1 YFP mice (Jackson Laboratory B6;CBA-Tg(Thy1-YFP)GJrs/GfngJ) were housed in standard conditions (20°C, 12/12 h light/dark cycle), with access to food and water *ad libitum*. Animals were monitored daily for signs of illness and stress and twice daily following surgery.

### Osmotic mini-pump preparation

Mini-pumps were prepared according to the protocol described in Sun and collegues with modifications (Sun et al., 2006). Pump preparation was performed in a sterile biohazard hood using aseptic techniques. To ensure appropriate placement of the catheter and focused delivery, the fluorescent anterograde tracer Fluoro-Ruby (Fluorochrome, LLC) was added to both vehicle control and excitotoxin solutions. Azlet® model 1004 mini-pumps (infusion rate of 0.11 μL per hour) were filled with 5 mM kainic acid (Sigma) and 0.01 μM Fluoro-Ruby in artificial cerebrospinal fluid (aCSF: 125 mM NaCl, 5 mM KCl, 10 mM D-glucose, 10 mM HEPES, 2 mM CaCl_2_, 2 mM MgSO_4_ pH 7.4; all from Sigma). At this infusion rate the pumps release 2.5 μg of kainic acid per 24 h, which is 200-fold less than that delivered systemically for seizure induction (Bouilleret et al., 1999; Schauwecker, 2012; Iyengar et al., 2015). Vehicle control pumps were loaded with 0.01 μM Fluoro-Ruby and an equal total volume of aCSF. Polyurethane intrathecal catheters (0.23 mm outside diameter × 0.09 inside diameter, Alzet®) were removed from their sterile packaging and two lines 3 mm apart were marked on the distal end with a surgical skin marker pen (Secure-line™), to assist with later surgical placement of the catheter, and the Teflon coated stylet wire removed. Catheters were loaded with the kainic acid or vehicle control solution and attached to the mini-pumps, being careful to ensure there were no bubbles in the catheter. Pumps, with catheters attached, were then placed into 50 mL centrifuge tubes (Sigma) and tubes filled with 0.9% sterile saline, pH 7.4 and placed into a 37°C incubator for 48 h to ensure the pump was equilibrated and had started pumping.

### Surgery preparation

All surgical procedures were performed in a bioBUBBLE (Fort Collins co.) with 80–100 HEPA filtered air circulations per hour, designated for surgical procedures. Mice were transferred to the room, acclimatized for 30 min and weighed. The surgical bench and microscopes were wiped down with isopropanol alcohol (Isowipe®, Kimberly-Clark) and the area covered with autoclaved surgical drapes. Surgical instruments were also autoclaved. The osmotic pumps were transferred to a pre-warmed heat pad at 37°C. Mice were injected with a mixture of ketamine (120 mg/kg)/xylazine (10 mg/kg) and left in their cage on a heat pad until completely anesthetized (withdrawal reflexes checked using toe pinch). Once anesthetized, the mouse was removed from the cage, placed onto a heat pad, a small amount of ophthalmic ointment (Refresh® Lacri-lube®) was placed on each eyeball and the thoracolumbar area of the back, where the incision was to be made, was shaved and prepared for aseptic surgery.

### Osmotic mini-pump insertion

**Figure 1:**
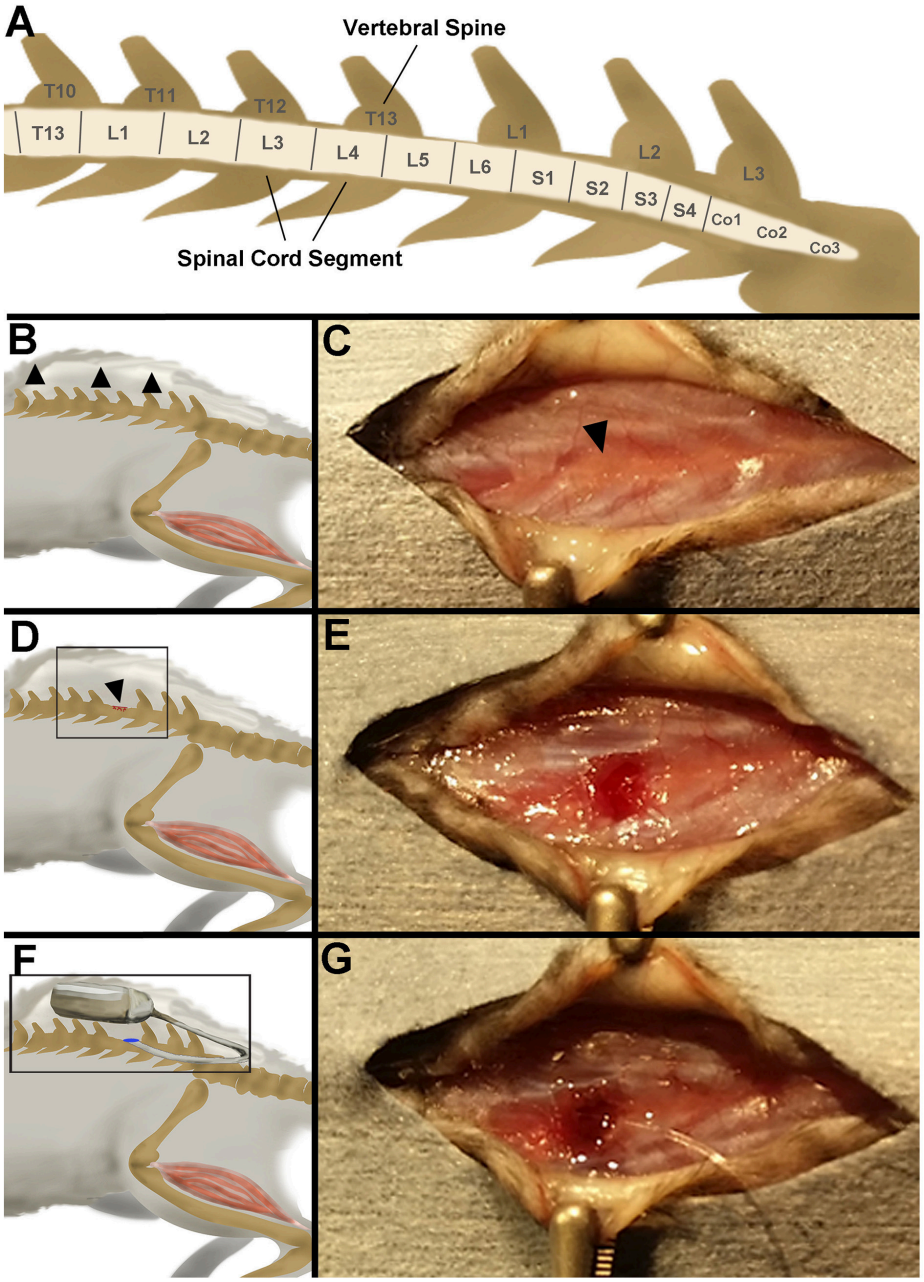
**(A)** Schematic of the spinal cord, referenced from MRI images of mouse C57BL/6J mouse (Harrison et al., 2013). **(B)** Schematic of the first procedure in the surgery—blunt dissection of skin pocket (arrowheads). **(C)** Step 1: a skin incision is made over the lumber region of the spinal cord and an anterior “pocket” is made under the skin for later placement of the pump. Arrowhead denotes T13. **(D)** Schematic of the second procedure in the surgery—vertebral spine dissection (arrowhead). **(E)** Step 2: the vertebral spine T13 is partially removed with micro-dissecting scissors. **(F)** Schematic of the third procedure in the surgery—catheter placement and pump insertion. **(G)** Step 3: The catheter is placed inside the subarachnoid cavity, the incision site secured with vetbond, and the pump placed in the skin pocket.

To achieve targeted delivery of excitotixin to the L3-4 lumbar region of the spinal cord the posterior portion of T13 vertebral spine (Figure 1A) was removed and catheter inserted 3 mm into the spinal column. A midline longitudinal incision in the mid-lumbar area was made with a size 10-scalpel blade. A subcutaneous pocket approximately 1.5 cm was blunt dissected along the spine to create an area for the pump to be placed (Figures 1B,C). Using forceps and micro scissors (Fine Science Tools), the posterior portion of the T13 vertebral spine (Figures 1D,E) was removed. Using a diamond edged goniotomy knife (Kaisers) the dura was gently pierced, being careful not to puncture the central blood vessel. Using fine tipped 45° angled forceps (Fine Science Tools) the pump catheter (which was cut at the first 3 mm mark from the distal tip) was inserted anteriorly into the subarachnoid space until the second 3 mm line is sitting in line with the partially removed vertebrae. The pump was gently squeezed with forceps, to check for resistance that would indicate blockage of the catheter. A 29-gauge needle was filled with Vetbond™ (3M™) and a drop carefully placed on top of the catheter and surrounding muscle tissue (Figure 1G). Once the glue had set, the pump was inserted in the subcutaneous pocket along the spine (Figures 1F,G). The incision site was closed with single interrupted sutures. A subcutaneous injection of Temegesic (0.01 mg/kg) was administered in 1 mL of pre-warmed saline. The mouse was then placed back in its home cage and the cage placed on a head pad until the mouse was fully alert. Subcutaneous Temegesic (0.01 mg/kg) was administered every 12 h for the first 48 h following surgery.

### Tissue processing

**Figure 2:**
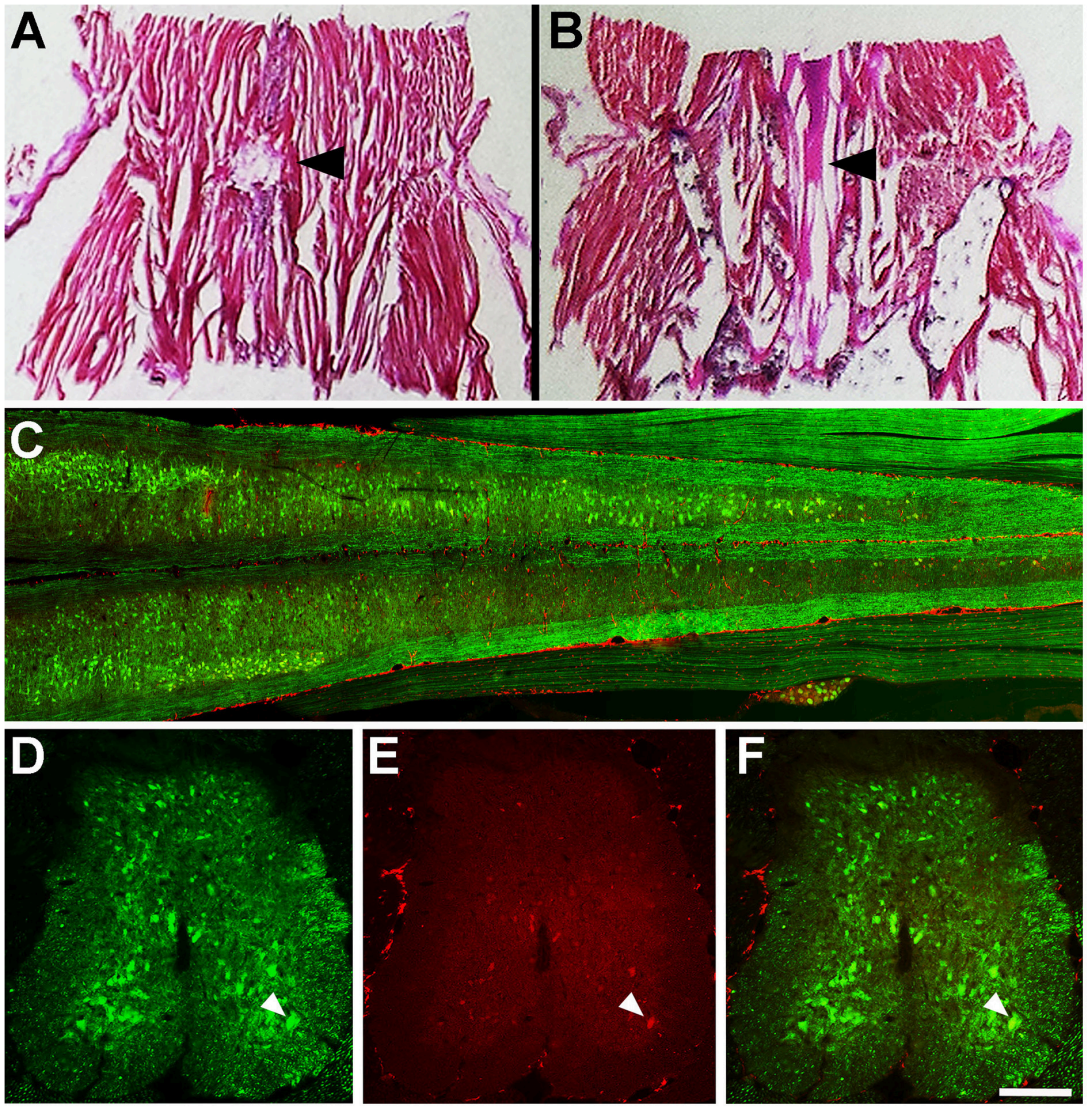
**(A)** H&E stained spinal column section at T13, superior to L5 where the catheter is inserted. Arrowhead indicates the incision at T13 and partial removel of veterbra. **(B)** L3-5 spinal cord section of the YFP mouse at 7 days infusion. Arrowhead indicates intact dorsal roots within the incision site. **(C)** Longitudinal section of L1-5 of the spinal cord (YFP, green) showing the location of Fluoro-Ruby (red) after 7 days infusion. **(D)** YFP (green) neurons in the L4 spinal cord at 7 days infusion **(E)**. Fluoro-Ruby (red) in the L4 spinal cord at 7 days infusion F. Merged image of L4 spinal cord YFP (green) and Fluoro-Ruby (red) at 7 days infusion. Arrowhead indicates a neuron in the anterior ventral horn that is positive for YFP **(D,F)** and Fluoro-Ruby **(E,F)**. Scale bar—**(A,B)** −3 mm, **(C)** −600 μm **(D–F)** 1500 μ m.

Mice were terminally anaesthetized (pentobarbitone sodium, 140 mg/kg) and perfused with 4% paraformaldehyde (Sigma) up to 28 days infusion. Spinal columns and gastrocnemius muscles were then dissected and left overnight in 4% paraformaldehyde. The L1 to L5 region of the spinal column was dissected and placed into a decalcification solution (5% nitric acid, 0.05% urea, both from Sigma, in MilliQ®) overnight. The spinal columns were then washed with 0.1M phosphate buffered saline, pH 7.4 (PBS), and placed into a series of overnight sucrose solutions (4, 18, and 30%, Sigma) in 0.1M PBS, 0.2% Sodium Azide (Sigma). Spinal cord sections (30 μm) were serially cut on a cryostat at −17°C and mounted onto superfrost™ plus slides (Thermo Scientific). For histology to investigate catheter placement (Figures 2A,B), L3-6 blocks were sectioned longitudinally in 40 μm sections and stained with Haemotoxylin and Eosin (H&E, Sigma). For immunohistochemistry spinal cord and sciatic nerve slides were incubated with primary antibodies (Anti-rat GFP IgG2a, 1:1000, Nacalai Tesque, Anti-mouse SMI-32 IgG, 1:2000, Covance) in 0.3% Triton-X (Sigma) in 0.1M PBS overnight, washed in 0.1M PBS and then incubated in secondary antibodies (All from Alexa-Flour®, 1:1000) for 90 min. SMI-32 is immunoreactive for non-phosphorylated neurofilaments, enriched in alpha motor neurons of the spinal ventral horn (Tsang et al., 2000). The GFP antibody is immunoreactive for green fluorescent proteins (GFP) and YFPs. Slides were then washed in 0.1M PBS, dried and coverslipped with a fluorescent mounting medium (DAKO). Gastrocnemius muscles were dissected, placed into a series of overnight sucrose solutions (4, 18, and 30%, Sigma) in 0.1M PBS for cryo-protection and sectioned at 80 μm. Muscle sections were stained with α-bungarotoxin (1:100; Molecular Probes®) in 0.1M PBS. Sciatic nerves were dissected placed into a series of overnight sucrose solutions (4, 18, and 30%, Sigma) in 0.1M PBS for cryo-protection and sectioned at 10 μm. All images were captured on a Zeiss LSM 510 confocal microscope using Zen software. Z-stacks were flattened using ImageJ (NIH) and processed in ImageJ and Photoshop (Adobe).

### Statistical analysis

The number of SMI-32 immunopositive neurons, with cell bodies greater than 20 μm in diameter, present in the anterior ventral horn in every second spinal cord section from L3 to L4 were counted (*n* = 4 for both vehicle control and 5 mM kainic acid animals). The number of intact and degenerating axons in the gastrocnemius muscle was determined by the colocalization of presynaptic YFP and post-synaptic α-bungarotoxin (intact) vs. the absence of the presynaptic YFP (degenerating). Student's *t*-tests were performed in GraphPad Prism Software, with *p* < 0.05 considered significant.

## Results

### Chronic delivery to the lumbar region of the mouse spinal cord can be achieved by an osmotic mini-pump and intrathecal catheter

Histological staining (H&E) of the mouse spinal column 1-day post-surgery demonstrated that using the described surgical approach resulted in a discreet laminectomy at T13 (Figures 1A, 2A), which left the dorsal roots close to the entry point of the catheter intact (Figure 2B). Fluoro-Ruby labeling was present throughout the subarachnoid space of the lumber region of the YFP spinal cord (Figure 2C). Furthermore, Fluoro-Ruby labeling was present in the anterior ventral horn (Figures 2D–F, arrowheads) confirming that the insertion of the catheter in the dorsal subarachnoid space leads to a dispersion of the fluid around the spinal column that reaches the ventral spinal cord and is taken up by neurons in this ventral region.

### Chronic kainic acid infusion to the L3-4 region of the mouse spinal cord causes motor neuron loss

**Figure 3:**
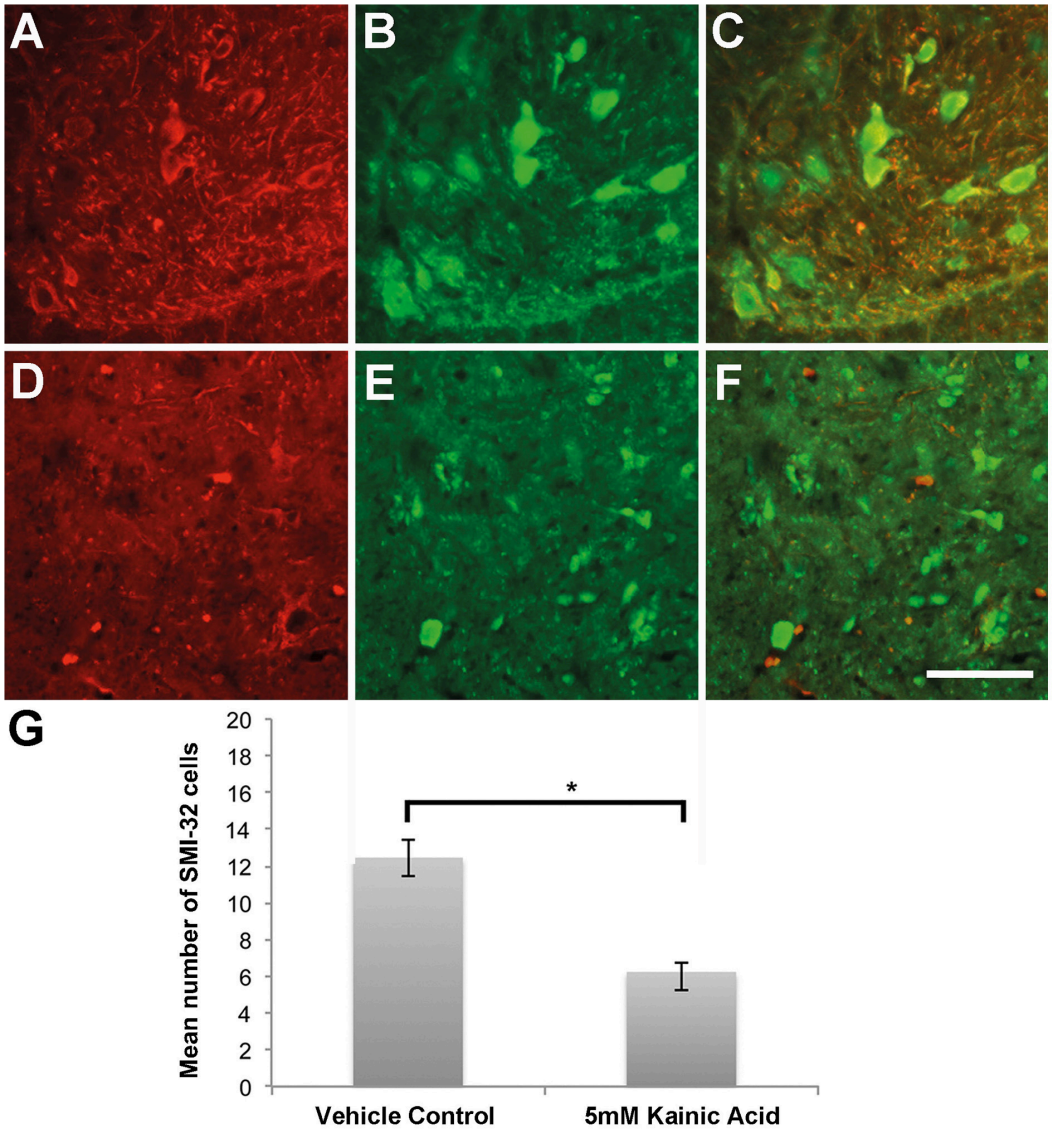
**(A,B)** Neurons within the anterior ventral horn of the L3-4 region of the spinal cord after 28 days infusion of vehicle control were immunoreactive for SMI-32 (**A** red) and YFP (**B** green). **(C)** Merged image. **(D,E)** Neurons within the anterior ventral horn of the L4 region of the spinal cord after 28 days infusion of 5 mM kainic acid had a shrunken morphology and decreased immunoreactivity for SMI-32 (**D** red) and YFP (**E** green). **(F)** Merged image. **(G)** Quantification of the number of SMI-32 positive neurons in the anterior ventral horn demonstrated a significant (*P* < 0.05) decrease after 28 days infusion of kainic acid in comparison to vehicle control. Scale bar −100 μm. ^*^P < 0.05.

Immunohistochemistry directed at endogenous YFP and SMI-32 demonstrated a loss of immunoreactivity in the anterior ventral horns after 28 days infusion with 5 mM kainic acid (Figures 3D–F), in comparison to vehicle control (Figures 3A–C). Quantitation of the number of cells immunopositive for SMI-32 demonstrated a significant (*P* < 0.05) decrease in the anterior ventral horn after 28 days infusion with 5 mM kainic acid in comparison to vehicle treated controls (Figure 3G).

### Chronic kainic acid infusion to the L3-4 region of the mouse spinal cord causes neuromuscular junction degeneration and axonal pathology

**Figure 4:**
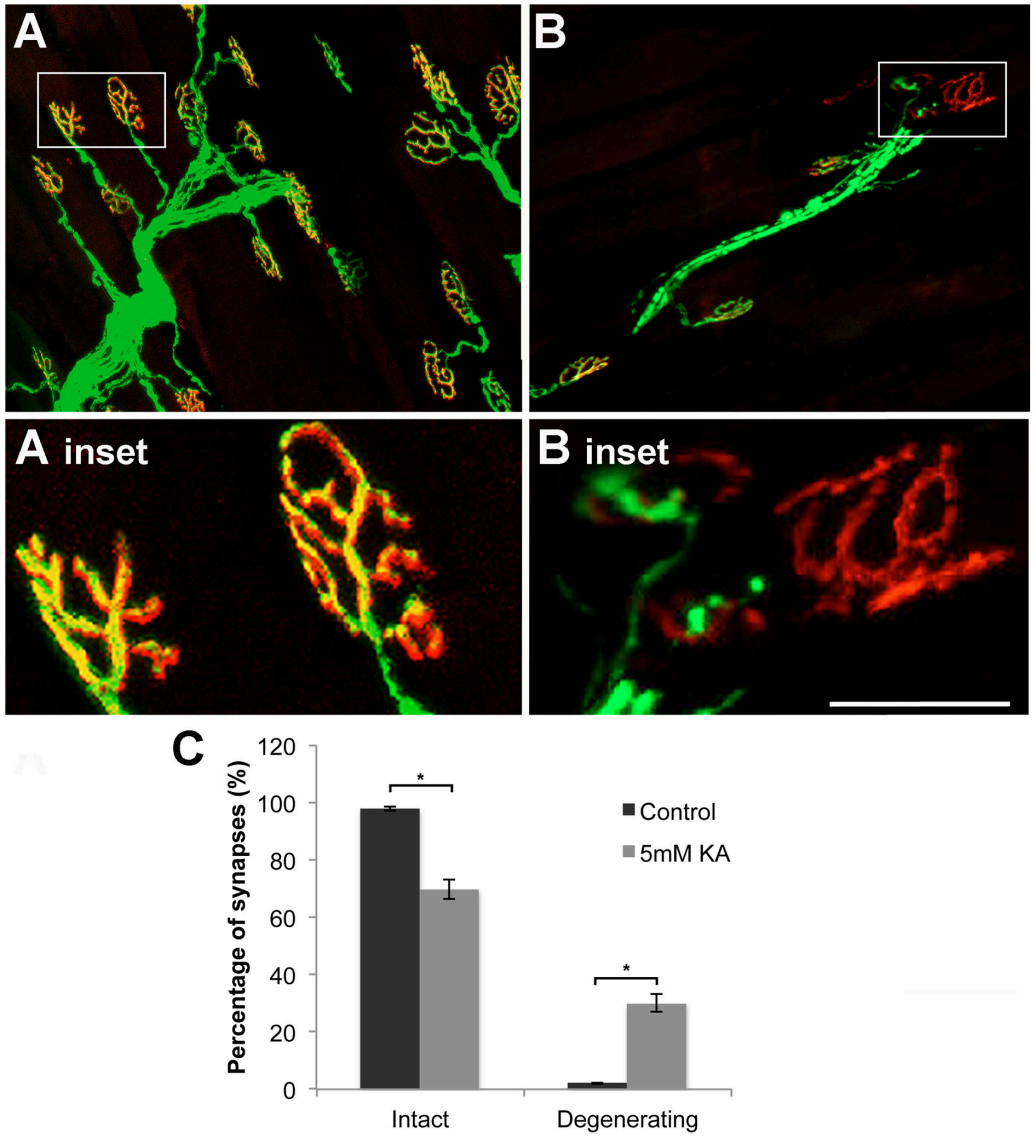
**(A)** After 28 days infusion with vehicle control, neuromuscular junctions, labeled for YFP (green) and α-bungarotoxin (red), were intact and showed classic morphologies (inset) throughout the gastrocnemius muscle. **(B)** After 28 days infusion with 5 mM kainic acid the neuromuscular junctions labeled for YFP (green) and α-bungarotoxin (red) were shrunken and the pre-synaptic terminal appeared retracted, leaving only α-bungarotoxin (red) intact (inset). **(C)** Quantitation of percentage of intact and degenerating synapses in the gastrocnemius muscle in vehicle control and 5 mM Kainic acid (KA) treated mice at 28 DPS demonstrated a significant (^*^P < 0.05) reduction in intact synapses and significant (^*^P < 0.05) increase in degenerating synapses. Scale bar—(**A,B)** –100 μm, **(A,B insets)** –50 μm.

**Figure 5:**
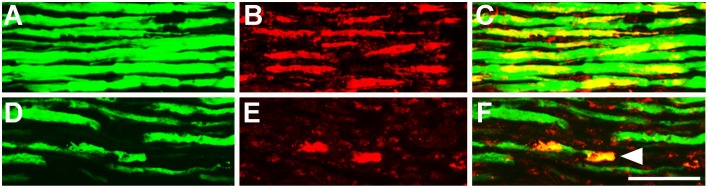
**(A–C)** Sciatic nerve section from vehicle control stained with YFP (**A** green) and SMI-32 (**B** red) at 28 days infusion demonstrated intact axons positive for both YFP and SMI-32 **(C)**. **(D)** Sciatic nerve section stained with YFP (**D** green) and SMI-32 (**E** red) after 28 days infusion of 5 mM kainic acid demonstrated a loss of immunoreactivity of SMI-32 with sporadic accumulations of SMI-32 in fragmented YFP^+^ axons (**F**, arrowheads). Scale bar –100 μm.

In the current model the gastrocnemius muscle was used to investigate neuromuscular junction pathology. Confocal microscopy stacks of neuromuscular junctions after 28 days infusion with vehicle control showed intact neuromuscular junction trees (Figure 4A) with complete colocalization between the pre-synaptic YFP and the post-synaptic stain α-bungarotoxin (Figure 4A inset). Following 28 days infusion of kainic acid (5 mM) the neuromuscular junction trees were frequently shrunken (Figure 4B). Staining with α-bungarotoxin revealed that whilst the post-synaptic terminal remains after 28 days infusion kainic acid (5 mM) the pre-synaptic YFP positive terminal had retracted (Figure 4B inset). Quantitation of the number of intact and degenerating synapses demonstrated a significant (*P* < 0.05) reduction in the percentage of intact synapses and a significant (*P* < 0.05) increase in the percentage of degenerating synapses in the 5 mM KA treated gastrocnemius muscles in comparison the vehicle control (Figure 4C). Investigations in the sciatic nerve demonstrated that after 28 days infusion axonal fragmentation of YFP positive axons could be observed in the kainic acid treated mice (Figures 5D,F) in comparison to vehicle control treated animals (Figures 5A,C). Immunohisotchemistry for the antibody directed at non-phosphorylated neurofilament, SMI-32, indicated that the YFP loss and fragmentation of axons in the KA treated sciatic nerve corresponded with a reduction in immunoreactivity for SMI-32 (Figures 5D–E). Furthermore, within the fragments of the degenerating axons, accumulations of SMI-32 were sporadically present (Figure 5F arrowhead).

## Discussion

Herein we describe the methods required to model excitotoxin mediated lower motor neuron death, and axonal and neuromuscular junction degeneration in the spinal cord, sciatic nerve and gastrocnemius muscle in the mouse. We demonstrated that, with the use of osmotic mini-pumps and intrathecal catheter attachments, the lumbar region of the mouse spinal cord can be specifically targeted without causing overt physical damage to surrounding areas. Furthermore, with the use of the YFP expressing transgenic mouse we demonstrate that chronic exposure of lower motor neuron cell bodies to the AMPA receptor agonist kainic acid leads to axonal and neuromuscular junction degeneration. This timely model will be of use to investigate lower motor neuron pathology related to neurodegenerative disease such as ALS. Indeed, ALS is characterized by the loss of both upper and lower motor neurons and the degeneration of the neuromuscular junction that is replicated in this model.

The current method allows investigation of the effect of kainic acid at the L3-4 region of the mouse spinal cord. The L3-4 region of the spinal cord was selected as the site for kainic acid delivery as in the mouse this is where the majority of the motor neurons innervating the gastrocnemius muscle reside (Mohan et al., 2014). Kainic acid is routinely used as an experimental AMPA receptor agonist (Kwak and Nakamura, 1995; Nottingham and Springer, 2003; Mazzone et al., 2010; Mitra et al., 2013). Previous studies in the rat have demonstrated that delivery of AMPA receptor agonists causes the death of motor neurons in the spinal cord in a strain dependent manner (Sun et al., 2006). Specifically there was a significant loss in the number of motor neurons in the L5 spinal cord by 4 weeks in the Fischer rats however this loss was not present until 8 weeks in the Wistar rats. Additionally they found that the neuron loss corresponded with reduced rotarod performance. In the current mouse model, we found a significant loss of SMI-32 immunopositive neurons in the anterior ventral horn by 4 weeks (28 days infusion). Furthermore, previous investigations in our laboratory have demonstrated that this motor neuron loss also corresponds to significant hind limb functional deficits (Blizzard et al., 2015). Collectively our data demonstrate that AMPA mediated lower motor neuron death is recapitulated in the mouse model. Our data, showing an approximately 50% loss of large cells in the anterior ventral horn, in conjunction with previous research indicating no significant difference in cell loss at 28 days infusion between increasing doses of kainic acid (5–10 mM kainic acid) (Blizzard et al., 2015) suggests that there not all motor neurons in the anterior ventral horn of the mouse are vulnerable to excitotoxic damage, in the time frame investigated. Indeed, studies comparing populations of neurons that are vulnerable and resistant in ALS indicate that the susceptibility to ionotropic receptor alterations may confer neuronal survival in this disease (Brockington et al., 2013). Hence the current model of slow chronic excitotoxic death may not only be useful in investigating the neurons that are vulnerable to excitotoxicity but also for determining the morphological and biochemical characteristics that render motor neuron subpopulations resistant.

The current model demonstrates that chronic lower motor neuron excitotoxicity directly causes distal axonal pathology and retraction of the pre-synaptic terminals of the neuromuscular junction. Distal axonal alterations in lower motor neurons are an early pathological mechanism occurring both in human ALS (Fischer et al., 2004) and mouse models of motor neuron diseases (Gillingwater and Ribchester, 2003; Moloney et al., 2014). A common pathogenic consequence of ALS is disruption to the axonal cytoskeleton, specifically the neurofilaments (Van Damme et al., 2005). Interestingly, chronic excitotoxic spinal cord infusion leads to a disruption in the phosphorylation dependent neurofilament heavy chain marker SMI-32 distally, in the sciatic nerve. Understanding how a proximal signal can lead to distal pathology is vital in understanding the pathogenesis of ALS as there is a desperate need to slow down or stop the characteristic progressive neurodegeneration. Furthermore, the gastrocnemius muscle is the largest muscle in the hind limb of the mouse and is a mixed fiber type muscle (Bloemberg and Quadrilatero, 2012) enabling the potential for not only neuromuscular junction degeneration to be investigated but also motor neuron firing type vulnerability.

Transgenic mice expressing YFP, first described in Feng et al. (2000) have proved to be a valuable tool when modeling disease, specifically when investigating axon degeneration. Previous studies have used these mice to study axonal degeneration in many models, including experimentally induced autoimmune encephalomyelitis (Bannerman et al., 2005; Bannerman and Hahn, 2007); ischemic injury in cortical slices (McCarran and Goldberg, 2007), retinal ganglion cells following kainic acid injection (Massoll et al., 2013), optic nerve crush (Lindsey et al., 2015), and peripheral nerve injury (Groves et al., 2005). Herein, we describe a method for inducing lower motor neuron axonal degeneration chronically with an excitotoxin over a 28 days time course in the spinal cord *in vivo*, this chronic evolution of pathology being relevant to a range of neurodegenerative diseases that manifest in the aging nervous system. Furthermore, due to the design of the insertion these methods could also be applied to the site-specific infusion of therapeutics, viral DNA constructs and or anterograde and retrograde tracers.

This model recapitulates the characteristic pathology associated with excitotoxic damage to the spinal cord in rats and affords distinct advantages over previous models. Use of the mouse has demonstrated that motor neuron subpopulations are differentially vulnerable to chronic excitotoxicity. Use of the YFP mouse allows the investigation into the mechanisms of how chronic excitotoxicity leads to axonal and neuromuscular junction pathology. Finally, establishment of a model that recapitulates the sporadic form of ALS will be beneficial for the discovery of therapeutic interventions that are not reliant upon the currently identified genetic mutations.

## Author contributions

CB contributed to the design of experiments, performed all surgeries and wrote the manuscript. KL performed the majority of tissue processing. TD designed experiments and contributed to the writing of the manuscript.

### Conflict of interest statement

The authors declare that the research was conducted in the absence of any commercial or financial relationships that could be construed as a potential conflict of interest.
